# A simple surface plasmon resonance biosensor for detection of PML/RARα based on heterogeneous fusion gene-triggered nonlinear hybridization chain reaction

**DOI:** 10.1038/s41598-017-14361-5

**Published:** 2017-10-25

**Authors:** Bin Guo, Wei Cheng, Yongjie Xu, Xiaoyan Zhou, Xinmin Li, Xiaojuan Ding, Shijia Ding

**Affiliations:** 1 Key Laboratory of Clinical Laboratory Diagnostics (Ministry of Education), College of Laboratory Medicine, Chongqing Medical University, Chongqing, 400016 China; 2 The Center for Clinical Molecular Medical Detection, The First Affiliated Hospital of Chongqing Medical University, Chongqing, 400016 China; 3 Department of Clinical Laboratory, The Affiliated Hospital of North Sichuan Medical College, Nanchong, 637000 China

## Abstract

In this work, a simple and enzyme-free surface plasmon resonance (SPR) biosensing strategy has been developed for highly sensitive detection of two major PML/RARα (promyelocytic leukemia, retinoic acid receptor alpha) subtypes based on the heterogeneous fusion gene-triggered nonlinear hybridization chain reaction (HCR). On the gold chip surface, the cascade self-assembly process is triggered after the introduction of PML/RARα. The different fragments of PML/RARα can specifically hybridize with capture probes (Cp) immobilized on the chip and the hybridization DNA_1_ (H_1_). Then, the nonlinear HCR is initiated by the complex of Cp-PML/RARα-H_1_ with the introduction of two hybridization DNA chains (H_2_ and H_3_). As a result, a dendritic nanostructure is achieved on the surface of chip, leading to a significant SPR amplification signal owing to its high molecular weight. The developed method shows good specificity and high sensitivity with detection limit of 0.72 pM for “L” subtype and 0.65 pM for “S” subtype. Moreover, this method has been successfully applied for efficient identification of clinical positive and negative PCR samples of the PML/RARα subtype. Thus, this developed biosensing strategy presents a potential platform for analysis of fusion gene and early diagnosis of clinical disease.

## Introduction

PML/RARα (promyelocytic leukemia, retinoic acid receptor alpha) is the product of the chromosomal translocation t (15; 17) (q22; q21) and specifically occurs in acute promyelocytic leukemia (APL)^1,2^. Depending on the location of breakpoint, two major transcript subtypes of PML/RARα defined as long (L or bcr1) and short (S or bcr3) play an important role in APL development^3^. Therefore, efficient detection of the PML/RARα can provide the molecular basis for diagnosing and monitoring disease in APL patients.

Some conventional methods have been reported for detection of PML/RARα, such as flow cytometry^4^, real-time quantitative reverse transcription PCR^5^, chromosome analysis^6^, fluorescence *in situ* hybridization^7,8^ and microarray-based techniques^9^, etc. However, these techniques suffer from the intrinsic limitations of complicated preparation, low specificity and expensive reagent^10,11^.

To overcome these drawbacks, different biosensing strategies for detection of PML/RARα have been developed, including enzyme-amplified electrochemical biosensor^12^, dual-probe electrochemical DNA biosensor based on the “Y” junction structure^13^, and electrochemical biosensor based on nanoporous gold electrode^14^, etc. Despite these enzymatic signal amplification strategies acquire obvious improvement of analytical performance, the results are susceptible to be influenced by the interference of enzyme, limiting their wide application^15,16^. Hence, the development of enzyme-free biosensing platform is urgently desired.

Surface plasmon resonance (SPR) biosensor, with the advantages of enzyme-free, label-free and real-time^17^, has been widely used in the field of biosensing. However, compared with the electrochemical^18^ or chemiluminescence platform^19^, the low sensitivity of SPR limits its practical application for detection of biomolecules due to inability to measure extreme small changes in refractive index^20^.

Aiming to further improve sensitivity of biosensors, different amplification strategies have been explored for detection of nucleic acid molecules^21^. Among these strategies, various sandwich assays based on nanomaterials have been used in SPR analysis of biomolecules due to the simplified steps and great effect of signal amplification, such as metal nanoparticles^22,23^. More importantly, enzyme-free DNA self-assembly also shows great potential in amplification^24–26^, including catalytic hairpin assembly^27^, hybridization chain reaction (HCR)^28^, nucleic acid tweezers^29^, DNA walkers^30^ and DNA machines^31,32^. Among these strategies, hybridization chain reaction introduced by Dirks and Pierce provides a general principle to initiate the assembly of DNA hairpins into nanowires by a triggering chain^28^. Based on the linear polymerization of DNA hairpins, Hsing’s group develops a hairpin-free nonlinear HCR system by using six hybridization chains, in which the product of reaction is dendritic nanostructure by the self-assembly of DNA^33,34^. Subsequently, Wang’s group reported a simple nonlinear HCR electrochemical strategy for ultrasensitive detection of DNA by using five hybridization chains^35^. Recently our group reported a nonlinear HCR SPR biosensing strategy by using six hybridization chains for sensitive detection of nucleic acid^36^. However, these strategies suffer from intrinsic problems of multi-step and time-consuming.

Herein, to simplify the number of DNA chains for nonlinear HCR reaction and facilitate the protocol of detection, a novel surface plasmon resonance biosensing strategy has been developed for detection of two major PML/RARα subtypes based on heterogeneous fusion gene-triggered nonlinear HCR. The dendritic nanostructure is formed on the chip surface by the nonlinear HCR, which leads to a significant SPR amplification signal. This method shows high sensitivity and good specificity. Moreover, the strategy has been successfully applied for identification of clinical positive and negative PCR samples of the PML/RARα subtype. Therefore, this developed SPR biosensing strategy has great potential application for detection of fusion gene and early diagnosis of clinical disease.

## Results and Discussion

### Principle of the biosensing strategy

**Figure 1:**
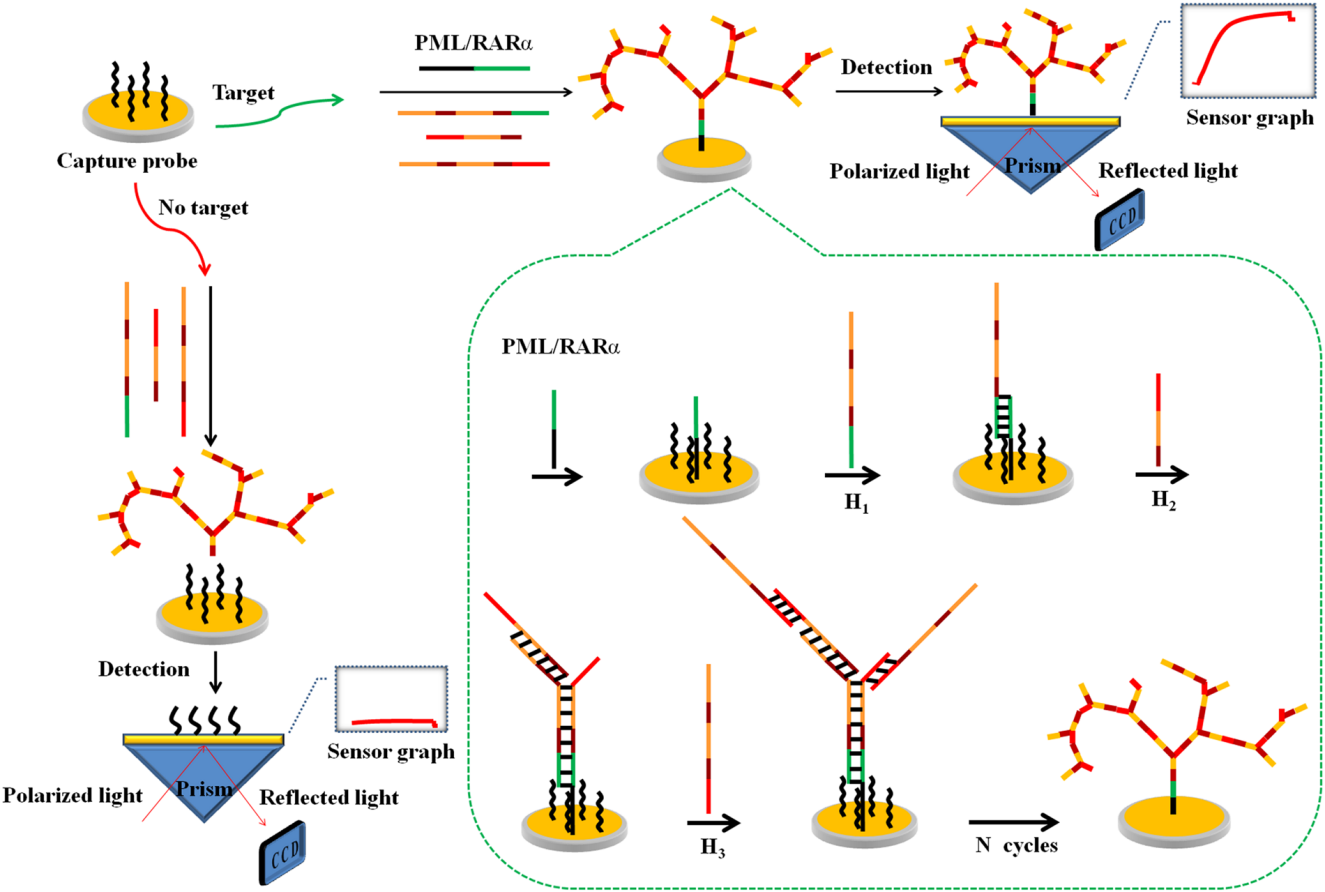
Schematic illustration of the SPR biosensing strategy via heterogeneous fusion gene-triggered nonlinear hybridization chain reaction for PML/RARα detection.

**Figure 2:**
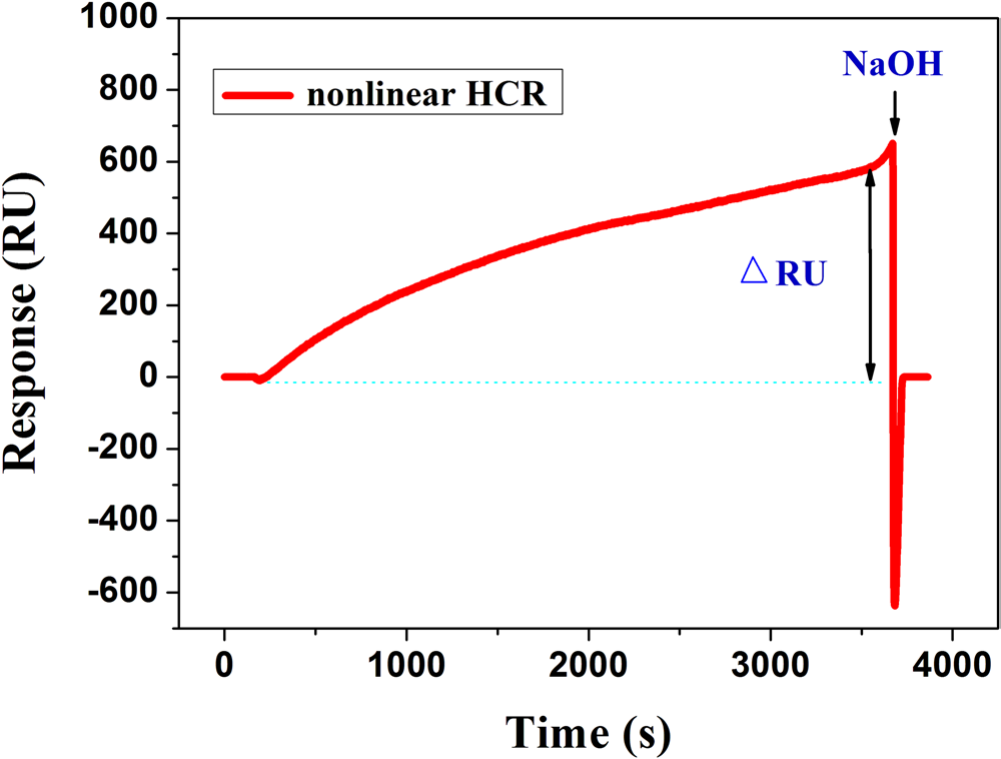
Typical SPR sensorgram of heterogeneous PML/RARα-triggered nonlinear hybridization chain reaction for signal amplification.

An overview of the designed SPR biosensing strategy for detection of PML/RARα subtype is illustrated in Fig. 1. Only four chains including Cp, H_1_, H_2_ and H_3_ are employed in this work. On the gold chip surface, the cascade self-assembly process is triggered after the introduction of PML/RARα used as linker. The 20 bases of PML fragment specifically hybridize with the Cp immobilized on the chip and the 20 bases of RARα fragment specifically hybridize with the H_1_, resulting in formation of the sandwich structure. Then, the H_1_ presents two identical sequences of series connection, which can simultaneously hybridize with two H_2_. Thus, there are two new exposed “toeholds” at end of each H_2_. Then, two H_3_ can hybridize with the “toeholds” respectively, and each H_3_ presents two identical sequences that complementary to H_2_. These hybridization chains are capable of initiating more rounds of similar hybridization reactions to “collect” more free H_2_ and H_3_ in the solution, resulting in dendritic growth of the DNA nanostructure on the gold chip surface. Thus, a high SPR signal is obtained owing to the high molecular weight of dendritic nanostructure. On the contrary, in the absence of the PML/RARα, the dendritic nanostructure exists only in homogeneous phase and almost negligible SPR signal is observed. Therefore, this study develops an one-step and enzyme-free SPR biosensing strategy for detection of PML/RARα subtype by using only four hybridization chains. A typical SPR sensorgram is depicted in Fig. 2, ΔRU shows the real time response of heterogeneous PML/RARα-triggered nonlinear HCR.

### Feasible analysis of the biosensing strategy

**Figure 3:**
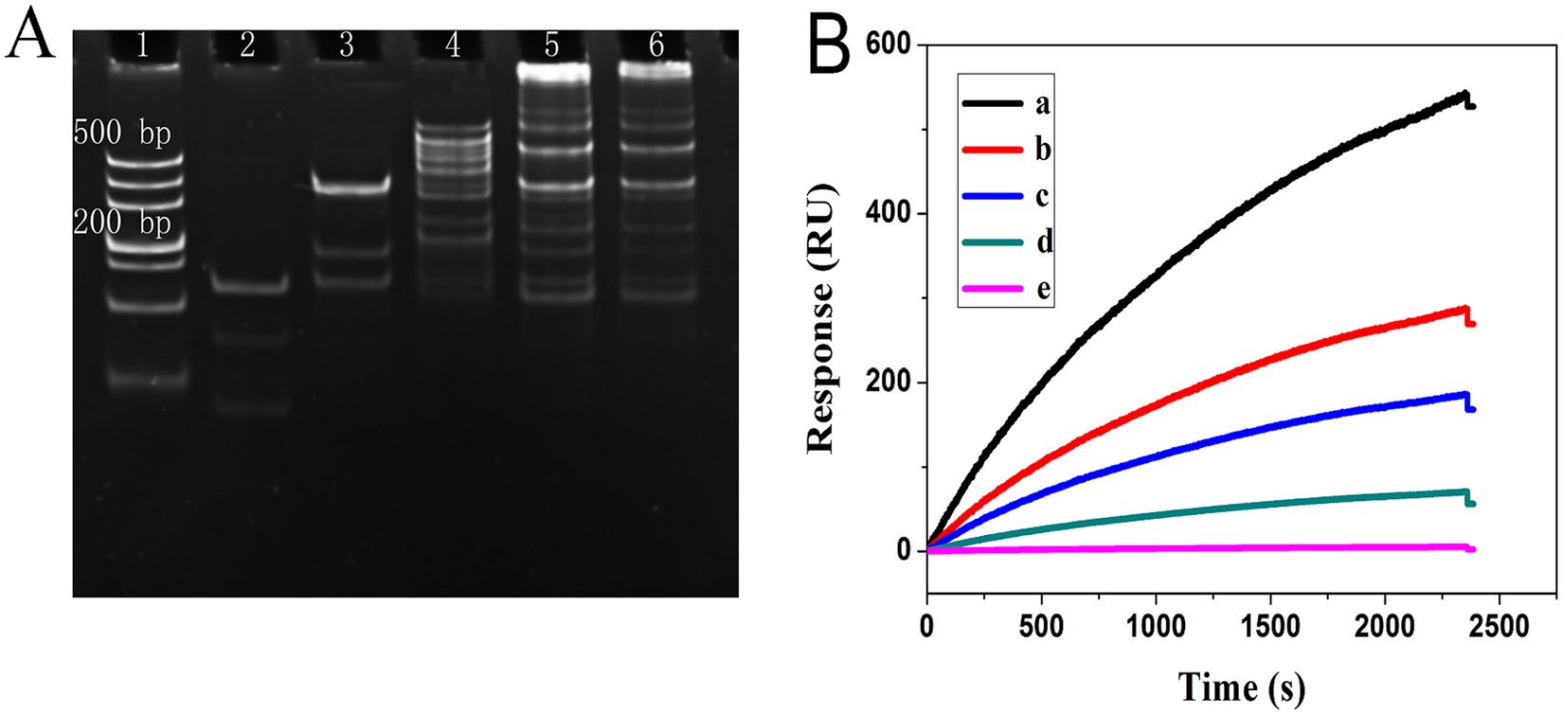
(**A**) 8% native polyacrylamide gel electrophoresis results of nonlinear hybridization chain reaction corresponding to hybridization of different chains: Lane 1: DL 500 DNA ladder marker; Lane 2: Cp and PML/RARα; Lane 3: Cp, PML/RARα and H_1_; Lane 4: Cp, PML/RARα, H_1_ and H_2_; Lane 5, 6: nonlinear hybridization chain reaction with 500 and 250 nM H_1_, H_2_ and H_3_, respectively. (**B**) SPR sensorgrams for different chains input corresponding to blank (curve e), PML/RARα (curve d), PML/RARα and H_1_ (curve c), PML/RARα, H_1_ and H_2_ (curve b), PML/RARα, H_1_, H_2_ and H_3_ (curve a).

To verify the validity of the nonlinear HCR amplification reaction, the 8% native polyacrylamide gel electrophoresis (PAGE) was performed by the hybridization of different chains. As shown in Fig. 3A, assembly products with high molecular weight were almost invisible with the hybridization of Cp and PML/RARα (Lane 2), Cp, PML/RARα and H_1_ (Lane 3). After the hybridization of Cp, PML/RARα, H_1_ and H_2_ (Lane 4), the bands with much lower mobility appeared, indicating the products with higher molecular weight were assembled. As anticipated, with the hybridization of Cp, PML/RARα, H_1_, H_2_ and H_3_ (Lane 5 and 6), the assembled products showed a broad distribution of size, and the average molecular weight was directly related to the concentration of hybridization chains (H_1_, H_2_ and H_3_) added. The above results indicated that the nonlinear HCR amplification reaction was feasible.

To further validate the feasibility of the developed HCR amplified strategy for detection of PML/RARα, SPR measurements were performed by the introduction of different chains. As shown in Fig. 3B, in the present of PML/RARα, high SPR signal was obtained because of the high molecular weight dendritic nanostructure assembled on the chip surface (curve a). On the contrary, almost negligible SPR signal was observed in the absence of PML/RARα (curve e), indicating the interaction could not occur between the capture probes and the hybridization DNA chains. For comparison, with only the introduction of PML/RARα, a small SPR signal was observed (curve d). After the additional introduction of H_1_ (curve c) and H_1_-H_2_ duplex (curve b), the SPR signals were further increased. However, these signals were much smaller than that obtained by the nonlinear HCR amplification reaction, demonstrating the feasibility of the designed SPR biosensing strategy.

### Optimization of experimental conditions

**Figure 4:**
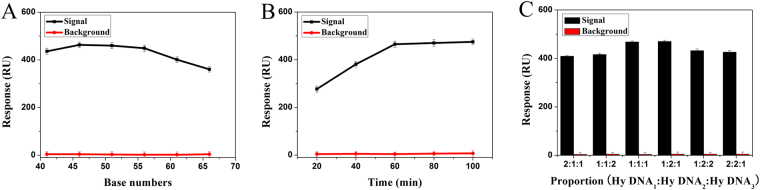
Optimization of experiment conditions. (**A**) evaluation of distance between the gold film and dendritic nanostructures. (**B**) evaluation of reaction time for the nonlinear hybridization chain. (**C**) evaluation of the proportion among H_1_, H_2_ and H_3_. Error bar represents the standard deviation (n = 3).

The experimental conditions were optimized to obtain excellent analytical performance. The distance between the gold film and the high molecular nanostructure has a great influence on SPR signal response and the DNA self-assembly efficiency. The SPR signal response takes place near the metal surface (0~200 nm) distance^21^. On the other hand, the self-assembly of dendritic nanostructure can be affected by the steric hindrance due to excessive close distance^31^. Hence, to obtain the best SPR response signal, different distances in the range from 41 to 66 bases were investigated in this work. As shown in Fig. 4A, the SPR signal-to-noise ratio started to increase from 46 bases distance and significantly decrease from 56 bases distance. The optimal signal-to-noise ratio was at 46 bases distance, which was chosen to further experiment.

To enhance sensitivity of detection, the reaction time was optimized. As shown in Fig. 4B, it was clear that the SPR signal increased with the augment of the reaction duration from 20 to 60 min, indicating more self-assembly products could be immobilized on the SPR chip surface. The optimal signal-to-noise ratio was observed when the reaction time was 60 min, and then the signal-to-noise ratio tended to stable after 60 min, indicating the strategy could not output great signal due to the SPR signal response distance and steric effect of self-assembly. Therefore, the reaction time of 60 min was selected.

In addition, to reduce the competitive interference, the proportion among hybridization chains was optimized, including H_1_: H_2_: H_3_ (2:1:1, 1:1:2, 1:1:1, 1:2:1, 1:2:2, 2:2:1). As shown in Fig. 4C, the SPR optimal signal-to-noise ratio was no significant difference between the proportion of 1:2:1 and 1:1:1, indicating the interference was identical under the condition of two proportions. However, the latter was selected to further experiment for cost-effective consideration.

### Analytical performance of the biosensing strategy

**Figure 5:**
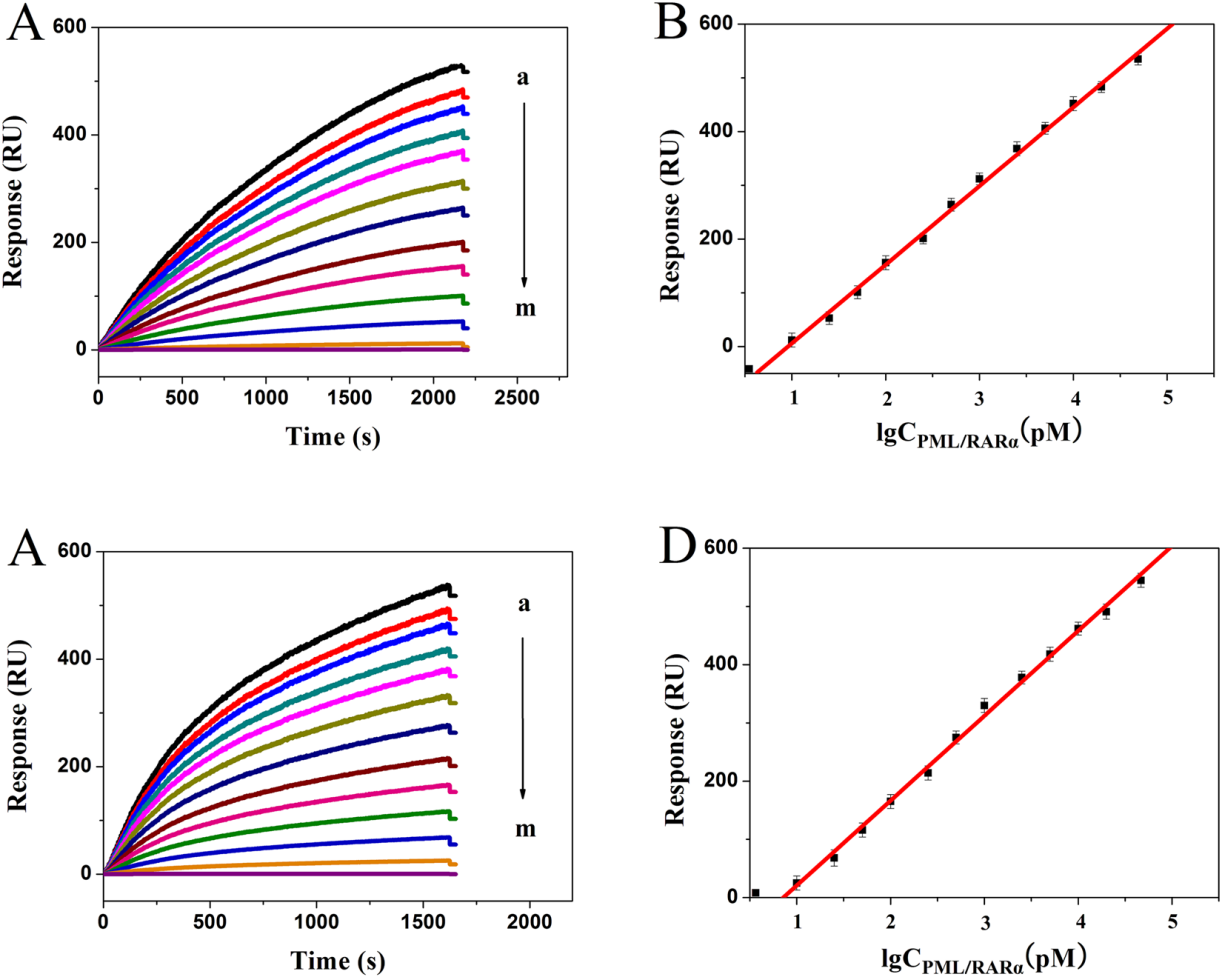
(**A** and **C**) represent respectively SPR sensorgrams for detection of PML/RARα with “L” and “S” subtypes at 50000, 25000, 10000, 5000, 2500, 1000, 500, 250, 100, 50, 25, 10, 0 pM (a to m). (**B** and **D**) represent respectively logarithmic plot of the designed strategy for detection of PML/RARα with “L” and “S” subtypes. Error bar represents the standard deviation (n = 3).

Under optimal experimental conditions, the intensity of SPR signal proportionally increased upon the introduction of different concentrations of PML/RARα DNA, ranging from 10 pM to50 nM (Fig. 5). The corresponding regression equations were Y = 146.41 × lgC (pM) − 140.56 with a correlation coefficient of 0.9983 for “L” subtype, and Y = 145.76 × lgC (pM) − 125.04 with a correlation coefficient of 0.9985 for “S” subtype. The detection limits estimated at 3σ were calculated to be 0.72 pM for “L” subtype and 0.65 pM for “S” subtype. The data were compared with those of reported SPR sensing methods (Table S2), demonstrating that this developed biosensor obtains the same great analysis performance with the merit of facilitated protocol, time-saving, and label-free.

### Specificity, reproducibility and recovery for PML/RARα detection

**Figure 6:**
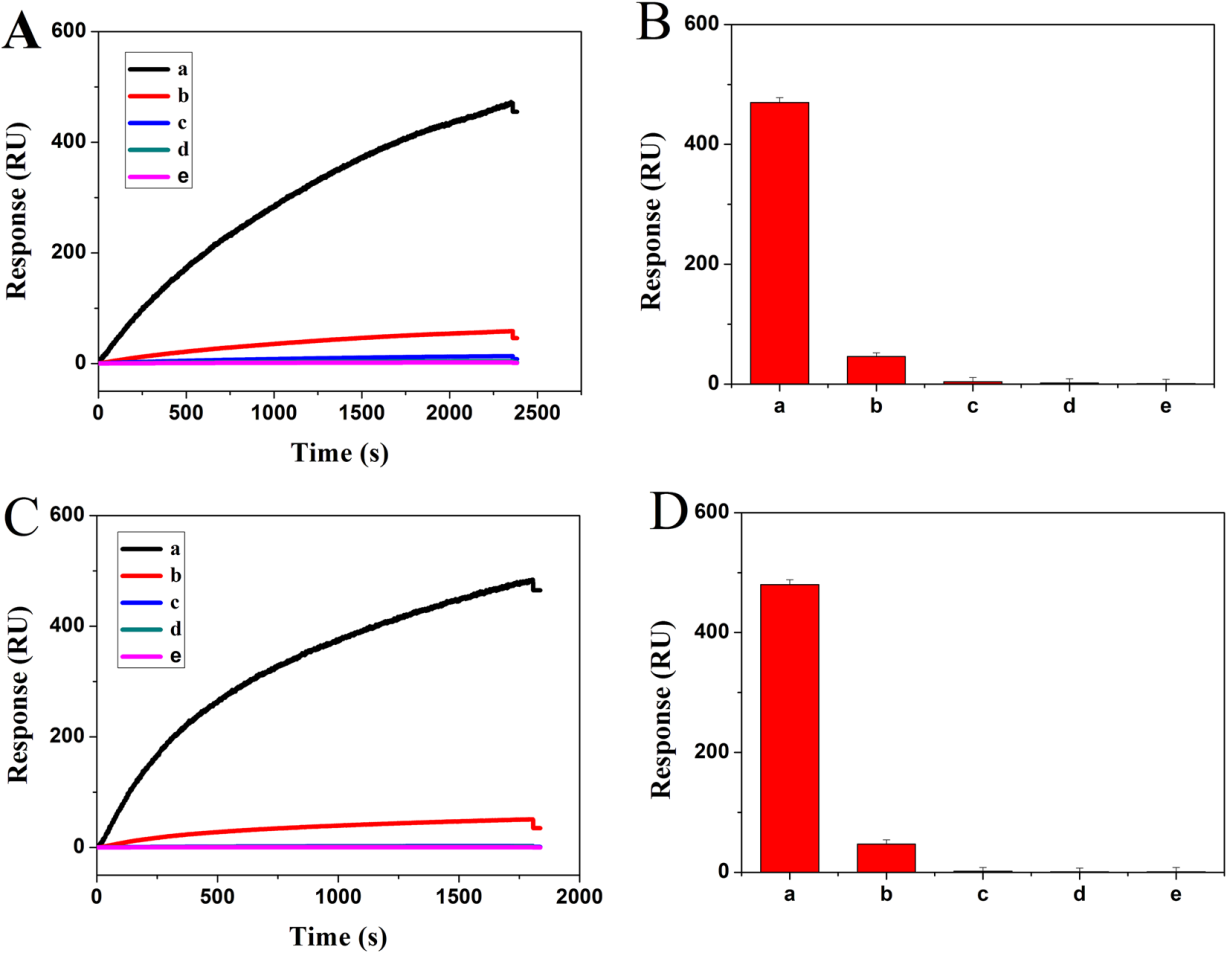
(**A**) SPR sensorgrams and (**B**) SPR response signal for specificity of “L” subtype corresponding to “L” subtype PML/RARα DNA (a), PML DNA 1 (b), “S” subtype PML/RARα DNA (c), RARα DNA (d), blank (e). (**C**) SPR sensorgrams and (**D**) SPR response signal for specificity of “S” subtype corresponding to “S” subtype PML/RARα DNA (a), PML DNA 2 (b), “L” subtype PML/RARα DNA (c), RARα DNA (d), blank (e). Error bar represents the standard deviation (n = 3).

A developed strategy was employed to evaluate specificity of the biosensor by detecting PML/RARα target DNA and three different DNA including PML DNA, RARα DNA and fusion gene DNA of different subtype (“L” or “S” subtype). As shown in Fig. 6, the addition of PML/RARα target DNA led to remarkable signal output (a). Nevertheless, in the presence of PML DNA, the SPR signal was only about 10% of that for PML/RARα target DNA (b). The SPR signal had no significant difference with the blank (e) in presence of RARα DNA (d) or fusion gene DNA of different subtype (c). Therefore, the fabricated biosensor showed good specificity due to the selective hybridization between Cp and target DNA.

In addition, five measurements on one chip for intra-reproducibility showed the coefficient of variations were 1.4% for “L” subtype and 1.0% for “S” subtype. Five measurements on different chips for inter-reproducibility showed the coefficient of variations were 3.3% for “L” subtype and 2.2% for “S” subtype (Table S3), indicating this biosensor had good reproducibility.

To verify the reliability of this developed biosensor in complex matrix, the recovery test was performed by using salmon sperm DNA. Samples of three different concentrations for two subtypes were prepared respectively by mixing synthetic PML/RARα DNA with 1 mg⋅mL^−1^ salmon sperm DNA. The recoveries were of 95–104% for “L” subtype and 98–105% for “S” subtype from 100 pM to 1000 pM (Table S4).

### Identification of PML/RARα subtype in real samples

**Figure 7:**
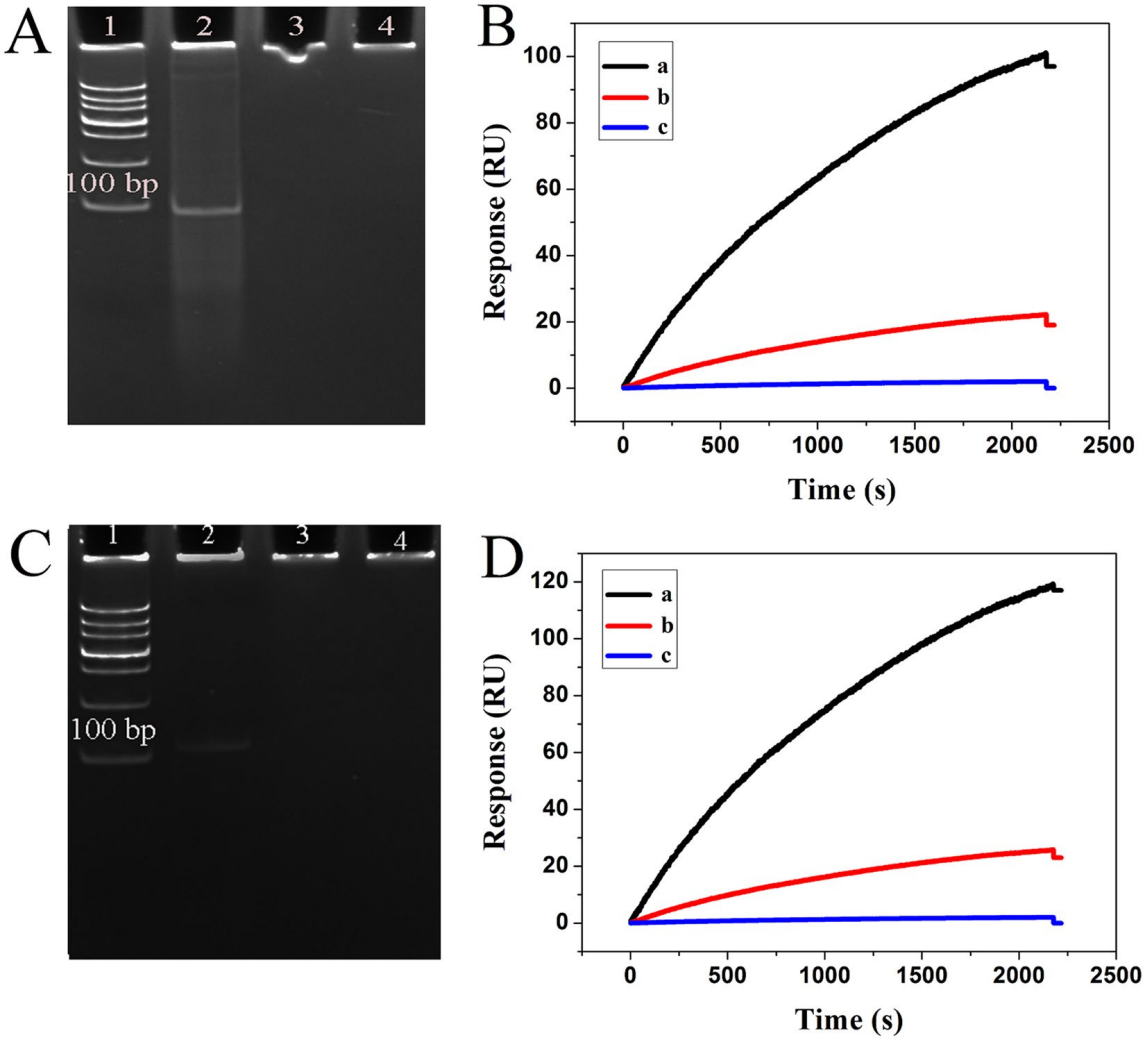
(**A** and **C**), the electropherogram of PCR products for “L” and “S” subtypes, respectively. Lane 1: DL 1000 DNA ladder marker; Lane 2: PML/RARα positive sample; Lane 3: PML/RARα negative sample; Lane 4: blank control. (**B** and **D**), SPR sensorgrams for real PCR samples with “L” and “S” subtype, respectively. The PML/RARα positive PCR sample (a), the PML/RARα negative PCR sample (b), the blank control (c).

To evaluate potential clinical application, the PCR amplification products were assayed by the developed biosensing method for detection of PML/RARα subtype. As shown in Fig. 7A,C, the PCR amplification products from the positive real sample showed the light band in lane 2. By contrast, no any band was observed in the gel with PCR products from negative real sample (lane 3) and blank control (lane 4).

The denatured PCR amplification products were detected by the developed biosensor. As shown in Fig. 7B,D, the small SPR signal was obtained as detecting the negative PCR samples (b), indicating the detection results could be influenced by non-specific hybridization due to the complex matrix effect. Interestingly, the high SPR signal was obtained as detecting the positive PCR samples (a), indicating the specific hybridization occurred between Cp and target DNA, despite the fact that products of PCR amplification were much longer than the synthetic target DNA fragments as described above. As shown in Fig. S1, the positive and negative real PCR samples gave a average value of 102 RU with the mean standard deviation (SD) of 5.12% and 21 RU with the mean SD of 3.61% for “L” subtype, and gave a average value of 122 RU with the mean SD of 6.48% and 19 RU with the mean SD of 2.74% for “S” subtype, indicating that the method had great identification of the clinical positive and negative PCR samples.

**Figure 8:**
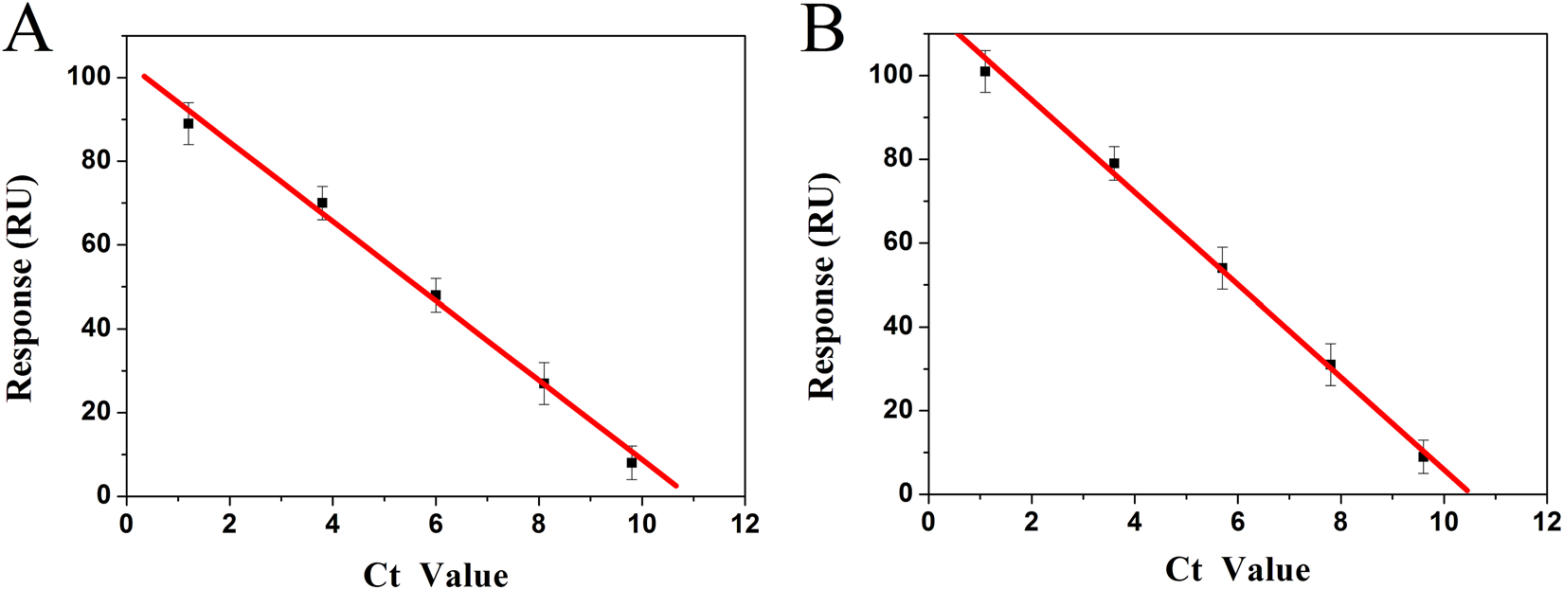
Comparison between the SPR signal of proposed biosensor and Ct value of qPCR assay for clinical PCR samples of different dilutions (10^2^, 10^3^, 10^4^, 10^5^, 10^6^) with “L” (**A**) and “S” (**B**) subtypes. Error bar represents the standard deviation (n = 3).

To further verify the detection accuracy of clinical PCR samples, real-time fluorescent quantitative PCR (qPCR) was used to measure the PCR products of different concentration. As comparing the results of two assays using regression analysis, the plots of cycle thresholds (Ct) obtained with the qPCR assay vs those of SPR signals obtained with the developed biosensor showed a good linear relationship with the correlation coefficient of 0.9966 for “L” subtype and 0.9983 for “S” subtype (Fig. 8), confirming the good consistency of two methods. On this basis, as the minimum △RUs based on SPR detection for clinical PCR samples were equivalent to the Ct value of 9.8 for “L” subtype and Ct value of 9.6 for “S” subtype based on qPCR assay, the LODs of the clinical PCR samples based on SPR detection were calculated to be 19.29 pM for “L” subtype and 18.09 pM for “S” subtype by the regression equation of qPCR assay (Y_Ct_ = 15.43–4.38 × lgC (pM) for “L” subtype, Y_Ct_ = 15.12–4.39 × lgC (pM) for “S” subtype) (Fig. S2). Therefore, practicality of the developed biosensor was further confirmed.

However, as the data shown, the SPR signals of clinical PCR samples were much smaller than those of synthetic samples. Two possible reasons contribute to the results. Firstly, long oligonucleotides may increase the possibility of forming secondary structure, which creates a higher energy barrier to intermolecular hybridization^37^. Secondly, a large number of nucleic acid from somatic cells can interfere with the efficiency of hybridization. These factors lead to the decrease of SPR signals. Therefore, the influence of secondary structure on kinetics of DNA hybridization should be further studied in the future.

## Conclusion

In summary, the present study has fabricated a simple SPR biosensing strategy for highly sensitive detection of two major PML/RARα subtypes based on heterogeneous fusion gene-triggered nonlinear HCR. This developed strategy not only improves the nonlinear HCR amplification reaction by using only four hybridization chains but also facilitates the protocol of detection. Undoubtedly, this strategy is time- and cost- saving without using any complex labels and enzymes. The method shows excellent sensitivity and specificity. Moreover, the method has efficient identification of the clinical positive and negative PCR samples. Therefore, as a optical platform, the developed SPR biosensing strategy with the advantages of real-time and enzyme-free has great potential application for detection of fusion gene in the field of laboratory medicine.

## Experimental

### Reagents and Materials

All HPLC-purified oligonucleotides were synthesized by Sangon Inc. (Shanghai, China), and the base sequences were listed in Table S1. All oligonucleotides were dissolved in hybridization buffer (pH 7.4) containing 30 mM sodium phosphate, 450 mM NaCl, 3 mM EDTA, and 0.25% Triton 100, and stored at −20 °C for further measurement. 6-Mercapto-1-hexanol (MCH) and salmon sperm DNA were purchased from Sigma-Aldrich (St Louis, MO, USA). RNA extraction Kit was purchased from Amoy Diagnostics (Xiamen, China). RT-PCR Kit was purchased from Yqbiomed (Shanghai, China). All other reagents were of analytical grade and without further purification. Ultrapure water from a Millipore water purification system (≥18 MΩ⋅cm, Milli-Q, Millipore) was used in all experiments.

### Apparatus

SPR experiments were measured with Biacore X™ analytical system (Biacore AB, Uppsala, Sweden) including a polarized light detection system, a sample-loading chamber, a micro-flow pump and a two-channel circulating detection cell. Sensing chips coated with gold film on the upside were obtained from Biacore AB. A time course of resonance units (RU) was employed to display the results. Sensorgrams were generated from the RU trace and were evaluated by fitting algorithms which compared the raw data to well-defined binding models. Cobas z analyzer was used for real-time fluorescent quantitative PCR (Roche, Switzerland). DYY-6C electrophoresis analyzer (Liuyi Instrument Company, China) and a Bio-rad ChemDoc XRS (Bio-Rad, USA) were used for gel electrophoresis and imaging.

### Preparation of SPR biosensor

The gold sensing chip pretreatment involved the following steps. First, the gold film was soaked in piranha solution (H_2_SO_4_: H_2_O_2_ = 3:1) for 10 min, followed by rinsing thoroughly with Millipore-Q water to eliminate other substances. The ready chip was docked in the Biacore X™ instrument socket and ran the sensorgram until a steady state baseline reached. Next, 1 μM thiolated capture probes in immobilization solution (1 mM KH_2_PO_4_, pH 3.8) were injected into the flow cell for 30 min. Third, the resulting chip was immersed into 1 mM MCH solution for 4 h to occupy the left bare sites on gold film and obtain well-aligned DNA monolayer. Finally, the chip was washed with Millipore-Q water, dried at room temperature and re-docked for further experiment.

### DNA analysis

One-step hybridization strategy was performed by simultaneous injection of PML/RARα (10 pM-100 nM), H_1_, H_2_ and H_3_ (100 nM) into the flow cell for 60 min. The sensing chips functionalized with different capture probes could be used for detection of two PML/RARα subtypes. Hybridization buffer was used as the running buffer in the experiment. All hybridization reactions were carried out at a flow rate of 1 μL⋅min^−1^. At the end of each cycle, the chip surface could be regenerated with 50 mM NaOH to remove the bound analytes on the gold film for the next detection. The target DNA was repeatedly measured by three times on the same chip and took the average value as the final result.

### Native polyacrylamide gel electrophoresis

The self-assembly of nonlinear HCR amplification reaction was ascertained by native polyacrylamide gel electrophoresis. All samples were electrophoresed on a 8% polyacrylamide gel (10% acrylamidein) in 1 × TBE (90 mM Tris-HCl, 90 mM boric acid, 2 mM EDTA, pH 7.9) buffer at constant voltage of 120 V for 26 min. The gel was stained with SYBR@Green II for 30 min and photographed by Bio-Rad digital.

### Identification of PML/RARα subtype in real samples

To verify the clinical applicability, the denatured PCR amplification products were assayed by the developed biosensing method for detection of PML/RARα subtype. All the PCR samples were obtained from the First Affiliated Hospital of Chongqing Medical University. First, bone marrow samples were collected and total RNA was extracted using TRIzol reagent. Then, to mix 2 μL Taq (2.5 U/μL), 6 μL reaction liquid (dNTPs, MgCl_2,_ Buffer), 1 μL sense primer (10 pmol/μL), 1 μL antisense primer (10 pmol/μL) and 15 μL sample into the reaction tube, the RT-PCR amplification protocol was as follows, 42 °C for 30 min, 5 min at 94 °C followed by 35 cycles of 94 °C for 45 s, 61 °C for 80 s, and 72 °C for 30 s. Then, the PCR products were characterized with 8% native polyacrylamide gel electrophoresis. Subsequently, all PCR samples were diluted by 10 times with hybridization buffer, denatured by 5 min at 95 °C and annealed by cooling the samples in an ice-water for 5 min. Finally, the positive PCR samples containing PML/RARα fragment and negative PCR samples were detected by the developed strategy, while using the buffer system as the blank control. After that, the clinical PCR samples were continuously diluted 10^2^, 10^3^, 10^4^, 10^5^, 10^6^ times for the SPR and qPCR detections.

## Electronic supplementary material

Supplementary information
