# Supplementary material for: A simple surface plasmon resonance biosensor for detection of PML/RARα based on heterogeneous fusion gene-triggered nonlinear hybridization chain reaction
Source: Sci Rep. 2017 Oct 25;7:14037. doi: 10.1038/s41598-017-14361-5 (PMC5656617; doi:10.1038/s41598-017-14361-5)
Supplement: Supplementary file 1 — Supplementary information [file 41598_2017_14361_MOESM1_ESM.doc]

**Supporting Information**

**A simple surface plasmon resonance biosensor for detection of PML/RARα based on heterogeneous fusion gene-triggered nonlinear hybridization chain reaction**

Bin Guo^a,c^, Wei Cheng^b^, Yongjie Xu^a^, Xiaoyan Zhou^a^, Xinmin Li^a^, Xiaojuan Ding^a^ & Shijia Ding^a^,*

*^a^Key Laboratory of Clinical Laboratory Diagnostics (Ministry of Education), College of Laboratory Medicine, Chongqing Medical University, Chongqing 400016, China*

*^b^The Center for Clinical Molecular Medical Detection, The First Affiliated Hospital of Chongqing Medical University, Chongqing 400016, China*

*^c^Department of Clinical Laboratory, The Affiliated Hospital of North Sichuan Medical College, Nanchong 637000, China*

**Corresponding author: Tel: +86-23-68485688, Fax: +86-23-68485786.*

*E-mail address: dingshijia@163.com (S.J. Ding) and* [*dingshijia@cqmu.edu.cn*](mailto:dingshijia@cqmu.edu.cn)*.*

**Table S1** Oligonucleotides employed in the present work

| Oligonucleotides^a^ | Sequence (5’-3’) ^b^ |
| --- | --- |
| Capture probe1 | CTGCCTCCCCGGCGCCACTG TTTTTT - (CH_2_)_6_-SH |
| Capture probe2 | CTTTCCCCTGGGTGATGCAA TTTTTT - (CH_2_)_6_-SH |
| H_1_ (61 bases) | *GTGTGCCTATTATGTCTCCTCCTGTGTGCCTATTATGTCTCCTCCT*TCTGGGTCTCAATGG |
| H_1_ (66 bases) | *GTGTGCCTATTATGTCTCCTCCTGTGTGCCTATTATGTCTCCTCCT*GCTGCTCTGGGTCTCAATGG |
| H_1_ (71 bases) | *GTGTGCCTATTATGTCTCCTCCTGTGTGCCTATTATGTCTCCTCCT*TTTTTGCTGCTCTGGGTCTCAATGG |
| H_1_ (76 bases) | *GTGTGCCTATTATGTCTCCTCCTGTGTGCCTATTATGTCTCCTCCT*TTTTTTTTTTGCTGCTCTGGGTCTCAATGG |
| H_1_ (81 bases) | *GTGTGCCTATTATGTCTCCTCCTGTGTGCCTATTATGTCTCCTCCT*TTTTTTTTTTTTTTTGCTGCTCTGGGTCTCAATGG |
| H_1_ (86 bases) | *GTGTGCCTATTATGTCTCCTCCTGTGTGCCTATTATGTCTCCTCCT*TTTTTTTTTTTTTTTTTTTTGCTGCTCTGGGTCTCAATGG |
| H_2_ | *AGGAGGAGACATAATAGGCACAC*TGACGAACTAGTTGATGAAGCTG |
| H_3_ | *GTGTGCCTATTATGTCTCCTCCTGTGTGCCTATTATGTCTCCTCCT*CAGCTTCATCAACTAGTTCGTCA |
| PML/RARα  Bcr1 (L) Target | CAGTGGCGCCGGGGAGGCAGCCATTGAGACCCAGAGCAGC |
| PML/RARα  Bcr3 (S) Target | TTGCATCACCCAGGGGAAAGCCATTGAGACCCAGAGCAGC |
| PML DNA 1 | CAGTGGCGCCGGGGAGGCAGGTAGGGAGAGGAACGCGTTG |
| PML DNA 2 | TTGCATCACCCAGGGGAAAGATGCAGCTGTATCCAAGAAA |
| RARα DNA | TAGCACACCATCCCCAGCCCCATTGAGACCCAGAGCAGC |
| Bcr1 (L) Primer |  |
| Sense | GGTCTTCCTGCCCAACAGC |
| Antisense | GGGCTGGGCACTATCTCTTC |
| Bcr3 (S) Primer |  |
| Sense | CGCACCGATGGCTTCGACG |
| Antisense | GGAGGGCTGGGCACTATCTC |

^a^ H_1_, hybridization DNA_1_; H_2_, hybridization DNA_2_; H_3_, hybridization DNA_3_.

^b^ The red portions represent complementary sequences between capture probe and PML DNA. The green portions represent complementary sequences between H_1_ and RARα DNA. The blue portions are used to control the distance between the gold film and the dendritic nanostructure. The underline portions represent complementary sequences between H_2_ and H_3_. The italic portions represent the sequences of H_2_ complementary to H_1_ and H_3_.

**Table S2** Comparison of different enzyme-free nucleic acid detection methods on Biacore X SPR biosensing platform

| Target | Strategy^a^ | Time | Dynamic range | LOD | Reference |
| --- | --- | --- | --- | --- | --- |
| miRNA | CHA and streptavidin aptamer | 90 min | 5 pM-100 nM | 1 pM | S1 |
| miRNA | MNAzyme-CHA | 180 min | 1 pM-100 nM | 1 pM | S2 |
| DNA | Multi step  nonlinear HCR | 90 min | 1 pM-1 nM | 0.85 pM | S3 |
| DNA | Hairpin capture  and streptavidin | 60 min | 10 pM-1 nM | 0.90 pM | S4 |
| DNA | One-step  nonlinear HCR | 60 min | 10 pM-50 nM | 0.72 pM  0.65 pM | This work |

^a^CHA, catalytic hairpin assembly; MNAzyme, multi component nucleic acid enzyme; HCR, hybridization chain reaction.

**Table S3** SPR signals for five replicate intra- and inter-measurements of 1 nM PML/RARα DNA

| Subtype | Group | Parallel tests | | | | | Average | SD^a^ | CV (%)^b^ |
| --- | --- | --- | --- | --- | --- | --- | --- | --- | --- |
| L | intra | 312.1 | 320.2 | 318.5 | 309.3 | 316.5 | 315.3 | 4.47 | 1.4 |
| L | inter | 321.7 | 312.2 | 322.4 | 304.2 | 332.6 | 318.2 | 10.63 | 3.3 |
| S | intra | 331.1 | 335.2 | 332.5 | 334.2 | 329.5 | 332.3 | 2.38 | 1.0 |
| S | inter | 329.5 | 325.1 | 330.5 | 339.3 | 343.6 | 333.4 | 7.49 | 2.2 |

^a^Standard deviation, ^b^Coefficient of variation.

**Table S4** The recoveries determined using the developed method via spiking synthetic DNA into salmon sperm DNA.

| Sample  no. | Spiking value  (pM) | Assayed value (pM) | | CV^a^ (%) | | Recovery (%) | |
| --- | --- | --- | --- | --- | --- | --- | --- |
|  |  | L  subtype | S  subtype | L  subtype | S  subtype | L  subtype | S  subtype |
| 1 | 100.0 | 95.2 | 105.1 | 4.8 | 5.1 | 95.2 | 105.1 |
| 2 | 500.0 | 516.3 | 492.5 | 3.2 | 1.5 | 103.2 | 98.5 |
| 3 | 1000.0 | 1040.4 | 1048.2 | 3.1 | 4.8 | 104.0 | 104.8 |

^a^ Coefficient of variation.


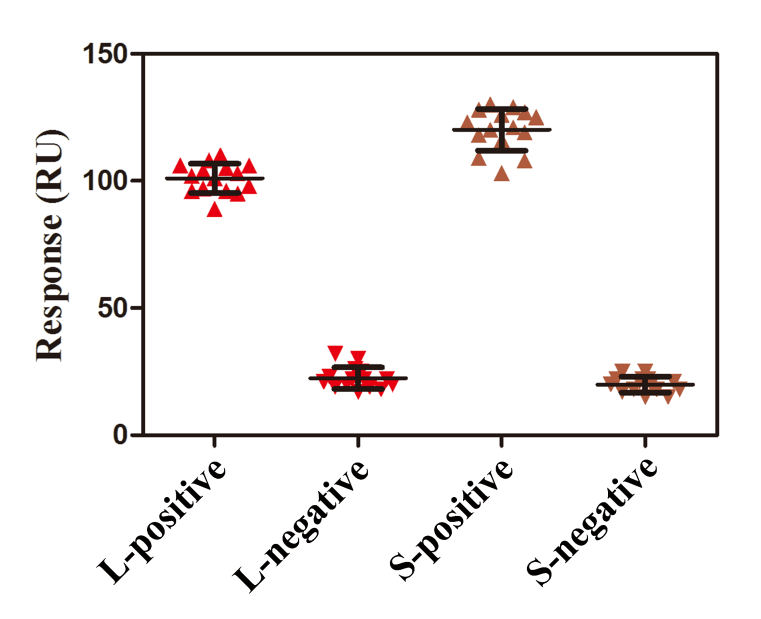


**Fig. S1** Comparison of SPR measurement data for PML/RARα positive and negative real samples with “L” and “S” subtypes

**
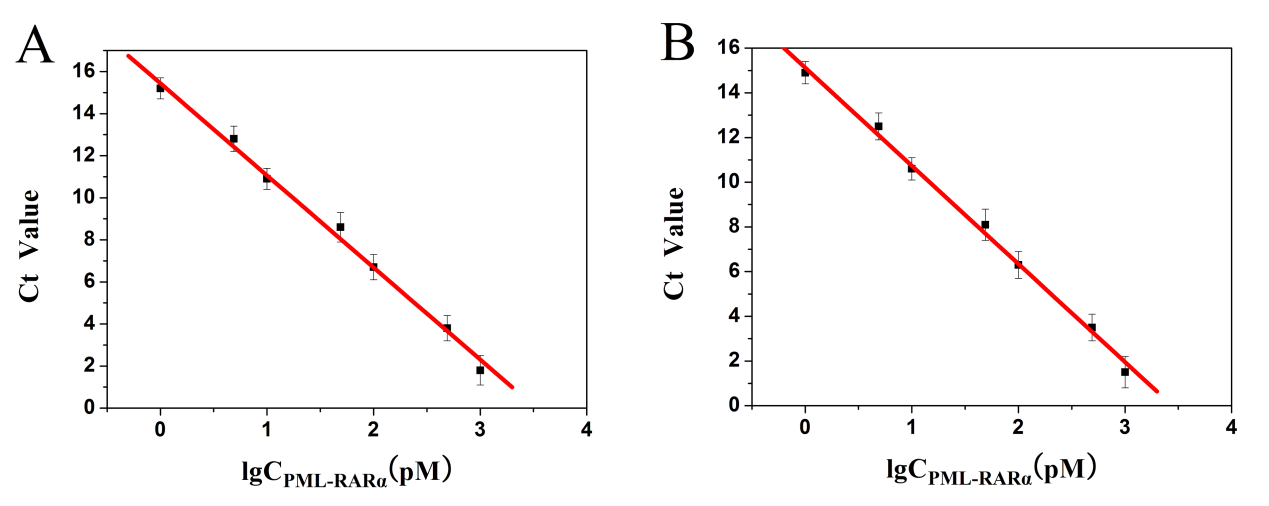
**

**Fig. S2** The standard curve of qPCR assay for “L” subtype (A) and “S” subtype (B) at 1000, 500, 100, 50, 10, 5, 1 pM. Error bar represents the standard deviation (n = 3)

**Reference**

# S1. Li, J.B., *et al.* An enzyme-free surface plasmon resonance biosensor for real-time detecting microRNA based on allosteric effect of mismatched catalytic hairpin assembly. *Biosensors and Bioelectronics* 77, 435-441 (2016).

S2. Li, X.M., *et al.* A novel surface plasmon resonance biosensor for enzyme-free and highly sensitive detection of microRNA based on multi component nucleic acid enzyme (MNAzyme)-mediated catalyzed hairpin assembly. *Biosensors and Bioelectronics* **80**, 98-104 (2016).

S3. Ding, X.J., *et al.* An enzyme-free surface plasmon resonance biosensing strategy for detection of DNA and small molecule based on nonlinear hybridization chain reaction. *Biosensors and Bioelectronics* **87**, 345-351 (2016).

# S4. Ding, X.J., *et al.* Surface plasmon resonance biosensor for highly sensitive detection of microRNA based on DNA super-sandwich assemblies and streptavidin signal amplification. *Analytica. Chimica. Acta.* 874, 59-65 (2015).
